# High genetic diversity of porcine rotavirus A, B, and C in Hungary with putative novel VP4 genotypes

**DOI:** 10.1186/s12917-026-05649-8

**Published:** 2026-06-19

**Authors:** Barbara Igriczi, Luca Zsiborás, Ervin Albert, Zoltán Német, Gyula Balka, Lilla Dénes

**Affiliations:** 1 Department of Pathology, University of Veterinary Medicine, István Str. 2, Budapest, 1078 Hungary; 2 National Laboratory of Infectious Animal Diseases, Antimicrobial Resistance, Veterinary Public Health and Food Chain Safety, University of Veterinary Medicine, István Str. 2, Budapest, 1078 Hungary; 3 Department of Pathology, University of Veterinary Medicine, Üllő, Hungary; 4 Centre for Metagenomics, Multidisciplinary Health Industry Coordination Institute, University of Debrecen, Debrecen, Hungary

**Keywords:** Porcine rotavirus, Genetic diversity, Epidemiology, Nanopore sequencing, Hungary

## Abstract

**Background:**

Rotaviruses (RVs) are important enteric pathogens of swine, contributing significantly to neonatal and post-weaning diarrhea worldwide. Although rotavirus A (RVA) is the best characterized species, much less is known about the epidemiology and genetic diversity of RVB and RVC, especially in Central Europe. This study aimed to investigate the presence and genetic diversity of RVA, RVB, and RVC in diarrheic piglets in Hungary using Nanopore third-generation sequencing.

**Results:**

A total of 77 fecal swab samples collected from diarrheic piglets across 19 swine farms were analyzed. All three RV species were detected, RVA and RVC were each identified in 54.5% of samples, while 40.3% was RVB positive. Coinfections involving multiple RV species were frequent, highlighting the complex etiology of piglet diarrhea. Altogether, 8 RVA, 3 RVB, and 4 RVC full-genome sequences, comprising all 11 segments, were identified. Genotyping of RVA strains revealed multiple G/P genotype combinations, with G9P[23] being the most prevalent. Whole-genome analysis demonstrated a Wa-like genomic backbone of porcine origin. In RVB, three complete VP4 sequences were obtained that could not be assigned to any known P genotype, suggesting the presence of a novel lineage. Hungarian RVC strains showed high genetic diversity, including five distinct G genotypes and one potential novel P genotype, underlining evolutionary diversity of porcine RVs.

**Conclusions:**

This study provides a comprehensive molecular characterization of RVA, RVB, and RVC circulating in Hungarian pig populations. The high prevalence of coinfections and the detection of genetically diverse and potentially novel strains emphasize the complexity of RV epidemiology in swine. These findings highlight the need for continued surveillance to better understand their role in pig health and zoonotic risk.

**Supplementary Information:**

The online version contains supplementary material available at https://doi.org/10.1186/s12917-026-05649-8.

## Background

Rotaviruses (RVs) belong to the *Sedoreoviridae* family and are significant pathogens associated with acute gastroenteritis in both animals and humans. The *Rotavirus* genus comprises nine species (A to J) classified based on the nucleotide sequence differences and antigenic properties of the inner capsid protein VP6, with two additional putative species (K and L) proposed more recently [1]. The RV genome consists of 11 segments of double-stranded RNA enclosed within a triple-layered particle, encoding six structural (VP1 to VP4, VP6, VP7) and five or six nonstructural proteins (NSP1 to NSP5/6) [2]. When observed under an electron microscope, RV particles typically display a characteristic wheel-like morphology [3].

A binary classification system, similar to that used for influenza viruses, categorizes the two outer capsid proteins, VP4 and VP7, both of which independently elicit neutralizing antibodies. Accordingly, RV strains are classified into VP4 (P, protease-sensitive) and VP7 (G, glycoprotein) genotypes [4]. As VP4 and VP7 are encoded by separate segments, reassortment following coinfection of a single cell can generate novel G/P antigen combinations. Several G and P genotypes are shared between RV strains infecting livestock and those detected in other hosts, including humans, horses, small ruminants, and birds, underscoring the zoonotic and interspecies transmission potential of RVs [5].

Among RV species, RVA (species *Rotavirus alphagastroenteritidis*) displays the highest genetic and antigenic diversity [6]. Currently, there are at least 42 G and 58 P genotypes recognized by the Rotavirus Classification Working Group (RCWG) [7]. In swine, at least 12 RVA, 22 RVB (species *Rotavirus betagastroenteritidis) *and 18 RVC (species *Rotavirus tritogastroenteritidis*) G genotypes, as well as 13 RVA, 2 RVB and 23 RVC P genotypes have been identified [8]. This diversity extends beyond the outer capsid proteins to all genome segments. For RVA, a comprehensive genotyping system based on nucleotide identity thresholds for each of the 11 segments has been established, using the genotype constellation format: Gx–P[x]–Ix–Rx–Cx–Mx–Ax–Nx–Tx–Ex–Hx, corresponding to the VP7–VP4–VP6–VP1–VP2–VP3–NSP1–NSP2–NSP3–NSP4–NSP5/6 genes [9]. For RVB and RVC, similar classification systems have been proposed, although a standardized application has not yet been established [10, 11]. Comparative genetic analyses indicate that the two major human RVA genotype constellations, the Wa-like (I1–R1–C1–M1–A1–N1–T1–E1–H1) and the DS-1-like (I2–R2–C2–M2–A2–N2–T2–E2–H2) genogroups, share common origins with porcine and bovine RVA strains, respectively [12, 13].

RVs are transmitted via the fecal-oral route and are commonly associated with enteric infections that may result in watery diarrhea. The disease primarily affects young animals, with susceptibility decreasing with age, probably due to physiological changes and/or immunity acquired through prior exposure [14]. RVs have been reported in piglets worldwide. In swine production, RV-associated enteric disease is an important cause of neonatal and post-weaning diarrhea and is associated with increased morbidity, reduced growth performance, and economic losses [15, 16]. To date, four RV species (RVA, RVB, RVC, and RVH) have been detected in fecal or intestinal samples from pigs of various ages, both with and without clinical signs of diarrhea.

RVA is the most prevalent and pathogenic RV species in humans and many animal hosts and is recognized as a leading cause of non-bacterial gastroenteritis worldwide. First identified in cattle in 1969 [17] and subsequently in children with acute gastroenteritis [18], RVA remains a major pathogen in neonatal and young pigs, although recent studies have also reported a high prevalence in post-weaning pigs, both in the presence and absence of diarrhea [19, 20]. RVB was initially detected in pigs with diarrhea [21] and has since been reported in several other mammalian hosts, including cattle, lambs and horses [22, 23]. Although frequently detected in coinfection with other RV species, certain outbreaks have implicated RVB as the primary causative agent. Epidemiological data indicate marked geographical variation in RVB prevalence, with higher detection rates reported in some parts of Asia and America [24–26], while generally lower rates in Europe, where the positivity is often higher in adult pigs [27–29]. RVC was first described in pigs in 1980 [30] and has since been detected in other species such as cattle, ferrets, dogs, and humans [31–34]. The presence of the virus has been reported worldwide [19, 29, 35], and RVC infection is commonly associated with diarrhea in suckling and weaned piglets [10], but its presence in post-weaning herds suggests ongoing transmission. Similar to RVA, recent genomic studies have revealed substantial genetic diversity within RVC also [36].

This study aimed to investigate the occurrence and genetic diversity of RVA, RVB, and RVC species in diarrheic piglets in Hungary, to identify the most prevalent G and P genotypes, and to determine complete genome sequences of selected strains.

## Results

### Initial RT-qPCR screening of the samples

Altogether, 77 samples were tested for RVA, RVB, and RVC by real-time reverse transcription PCR (RT-qPCR). A total of 62/77 samples (80.5%) were positive for at least one RV species. RVA and RVC were both detected in 42/77 cases (54.5%), while 31/77 (40.3%) samples were RVB positive. Coinfections were common, as 18 samples (31% of RV-positive samples) were positive for all three RV species, simultaneously. Dual infections were also identified, including RVA and RVC in 10 samples, RVA and RVB in 5 samples, and RVB and RVC in 2 samples. The distribution of single and mixed infections among the three RV species is summarized in Supplementary Figure S1. It should be noted that the prevalence of RVA may be partially overrepresented, as the fecal swab samples were pre-screened for RVA at the Livestock Diagnostic Center, and only those considered potentially suitable for Nanopore sequencing were forwarded to our laboratory for further analysis.

### Genetic analysis of Hungarian rotavirus A (RVA) strains

**Fig. 1:**
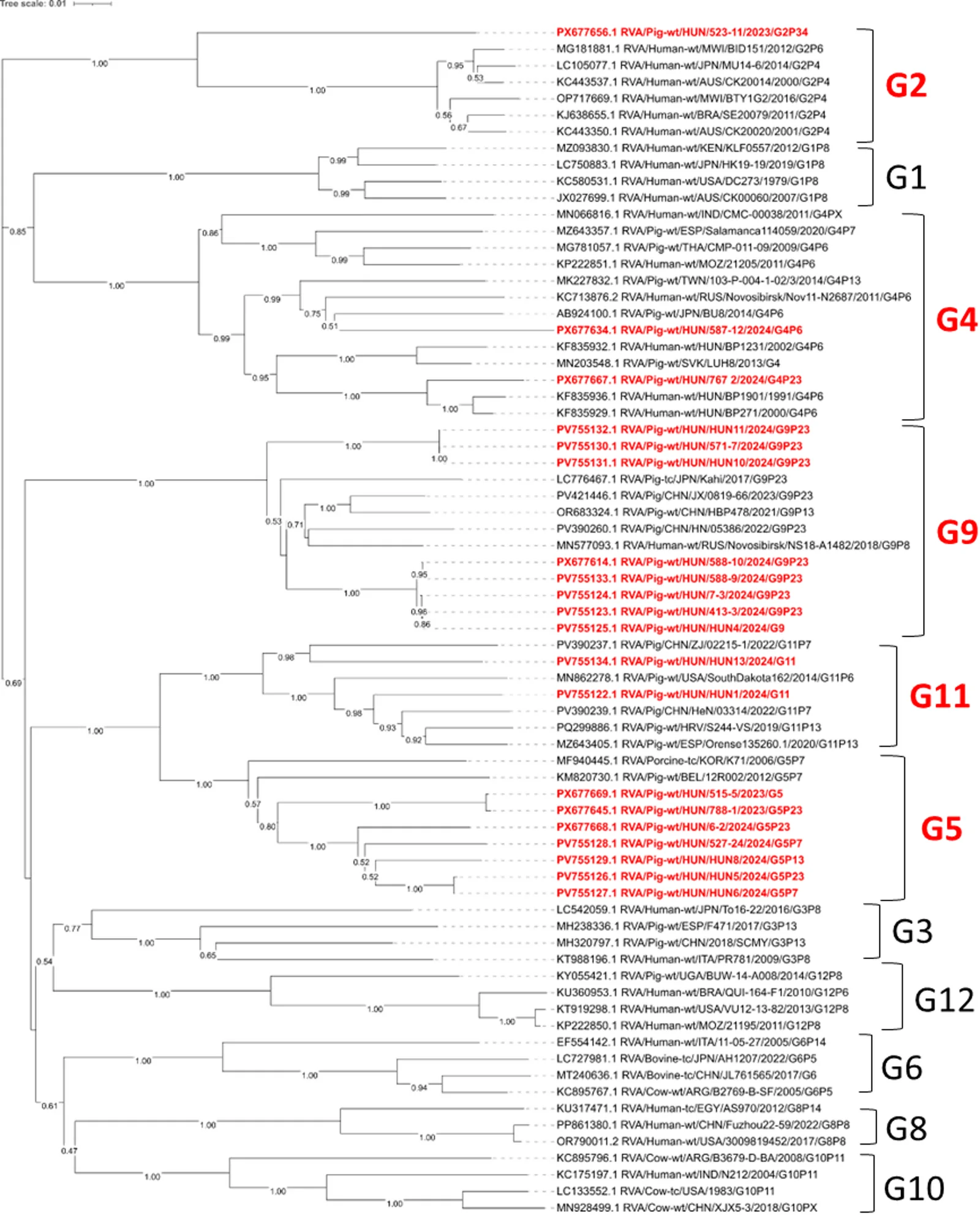
Phylogenetic analysis of the VP7 gene of rotavirus A (RVA). Hungarian RVA sequences are highlighted in red. G-genotypes are indicated on the right side of the tree. Phylogenetic analysis was conducted using MEGA X software with the neighbor-joining method, and node support was assessed using 1000 bootstrap replicates. Reference VP7 sequences were downloaded from GenBank and aligned with the Hungarian sequences to infer genetic relationships

A total of 20 complete VP7 sequences from Hungarian porcine RVA isolates were analyzed and classified into five G genotypes: G2 (*n =* 1), G4 (*n =* 2), G5 (*n =* 7), G9 (*n =* 8), and G11 (*n =* 2) (Fig. 1.). Phylogenetic analysis of the VP7 gene revealed that the G9 sequences formed two well-supported monophyletic clusters within the broader G9 clade, with bootstrap values > 99%. The nBLAST analysis of these G9 sequences revealed high similarity to Asian and Russian strains of human origin, indicating possible shared ancestry or past interspecies transmission. Intra-genotypic nucleotide identity within Hungarian G9 strains ranged from 90.93% to 100%, suggesting moderate genetic diversity. The G5 sequences were closely related to the Korean and Belgian porcine strains, with identities ranging from 88.58% to 99.9%, while the two G4 and G11 strains were more divergent, showing 86.22% and 86.14% nucleotide identity, respectively. Interestingly, one of the G4 sequences showed > 95% nucleotide identity to two Hungarian human RVA strains (KF835936.1 and KF835929.1) described in 2013, which had been isolated from diarrheic children [37]. The other G4 sequence exhibited lower identity values (85–89%) to these Hungarian human strains as well as to various porcine and human RVA strains of different origins. Although genotype G2 is frequently detected in human RVA infections, the single Hungarian G2 strain identified in this study showed the highest nucleotide similarity (84.34–93.78%) to porcine RVA reference strains, indicating a predominantly porcine origin.

**Fig. 2:**
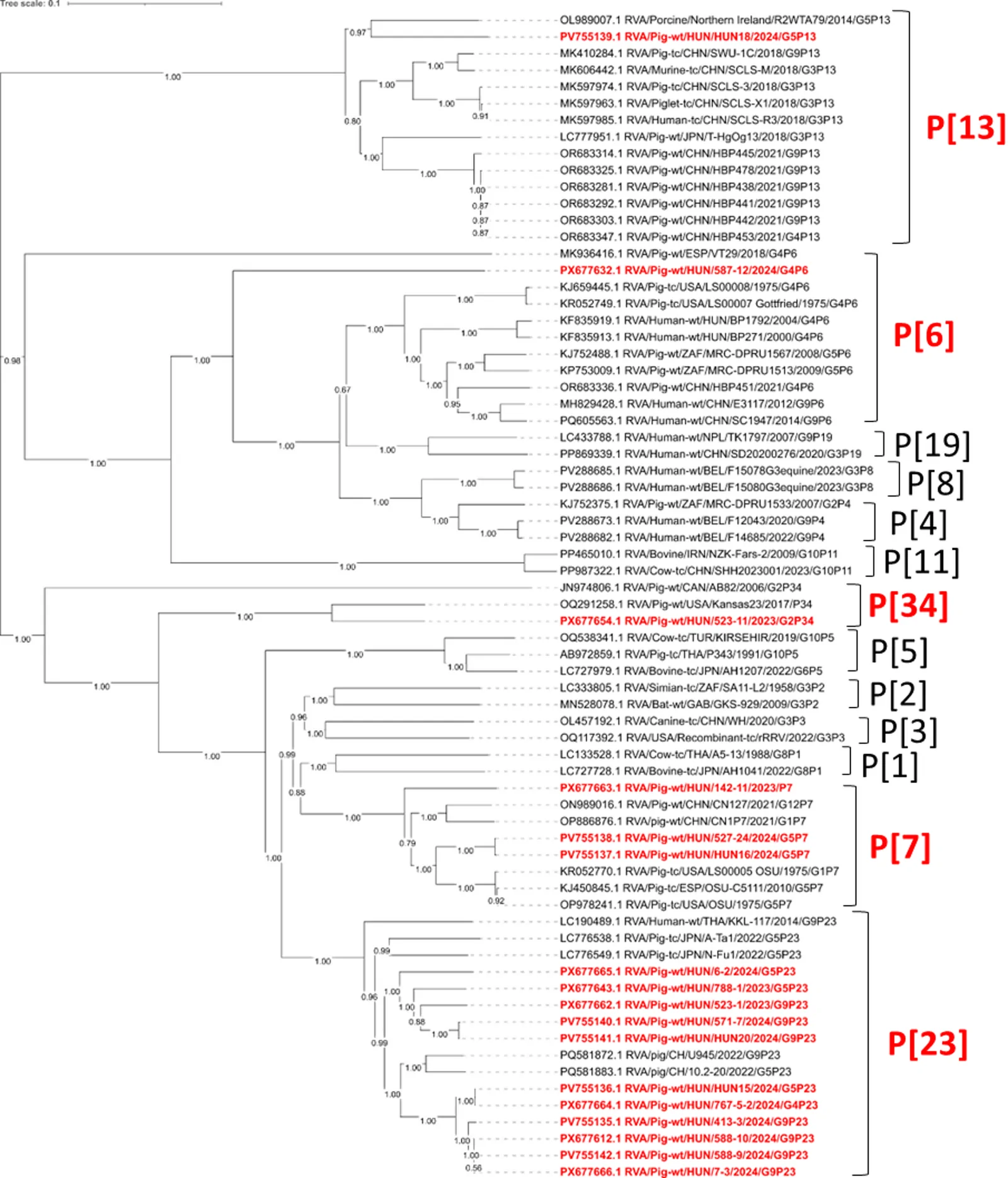
Phylogenetic analysis of the VP4 gene of rotavirus A (RVA). Hungarian RVA sequences are highlighted in red. P-genotypes are indicated on the right side of the tree. Phylogenetic analysis was conducted using MEGA X software with the neighbor-joining method, and node support was assessed using 1000 bootstrap replicates. Reference VP4 sequences were downloaded from GenBank and aligned with the Hungarian sequences to infer genetic relationships

For the VP4 gene, 17 complete sequences were analyzed, revealing five distinct genotypes: P[6] (*n =* 1), P[7] (*n =* 3), P[13] (*n =* 1), P[23] (*n =* 11) and P[34] (*n =* 1) (Fig. 2.). The P[23] sequences exhibited nucleotide identity ranging from 88.93% to 99.87%. The P[7] sequences showed high overall similarity: two nearly identical sequences (99.87% nucleotide identity) originated from the same farm, whereas a third P[7] sequence obtained from a geographically distant farm shared only approximately 88% nucleotide identity with them, indicating farm-level genetic clustering. The observed G/P combinations included: G9P[23] (*n =* 7), G5P[23] (*n =* 3), G5P[7] (*n =* 2), G5P[13] (*n =* 1), G4P[6] (*n =* 1), G4P[23] (*n =* 1) and G2P[34] (*n =* 1). Most of these strains clustered phylogenetically with porcine-derived sequences and represent G/P combinations frequently reported in pigs. An exception was the single P[6] strain, which appeared distinctly separated from porcine sequences in the phylogenetic tree. BLAST analysis indicated its closest genetic relatives (95.25–95.78% nucleotide identity) were two Hungarian human RVA strains (KF835919.1 and KF835913.1).

**Table 1:** Genome constellation of all Hungarian RV strains detected in this study

	Strain	VP7	VP4	VP6	VP1	VP2	VP3	NSP1	NSP2	NSP3	NSP4	NSP5	Genbank ID
RVA	RVA/Pig-wt/HUN/413-3/2024/G9P[23]	G9	P[23]	I5	R1	C1	M1	A8	N1	T1	E1	H1	PV755123, PV755135, PX677582-90,
	RVA/Pig-wt/HUN/527-24/2024/G5P[7]	G5	P[7]	I5	R1	C1	M1	A8	N1	T7	E1	H1	PV755128, PV755138, PX677591-99
	RVA/Pig-wt/HUN/571-7/2024/G9P[23]	G9	P[23]	I5	R1	C1	M1	A8	N1	T7	E1	H1	PV755130, PV755140, PX677620-28
	RVA/Pig-wt/HUN/588-9/2024/G9P[23]	G9	P[23]	I5	R1	C1	M1	A8	N1	T1	E1	H1	PV755133, PV755142, PX677600-08
	RVA/Pig-wt/HUN/588-10/2024/G9P[23]	G9	P[23]	I5	R1	C1	M1	A8	N1	T1	E1	H1	PX677609-19
	RVA/Pig-wt/HUN/587-12/2024/G4P[6]	G4	P[6]	I5	R1	C1	M1	A8	N1	T1	E1	H1	PX677629-39
	RVA/Pig-wt/HUN/788-1/2023/G5P[23]	G5	P[23]	I5	R1	C1	M1	A8	N1	T1	E1	H1	PX677640-50
	RVA/Pig-wt/HUN/523-11/2023/G2P[34]	G2	P[34]	I5	R1	C1	M1	A8	N1	T7	E9	H1	PX677651-61
RVB	RVB/Pig-wt/HUN/527-8/2024/G20P[X]	G20	P[X]	I11	R4	C4	M4	A8	N10	T4	E4	H7	PV755145, PX659544-53
	RVB/Pig-wt/HUN/142-7/2023/G6P[X]	G24	P[X]	I13	R4	C4	M4	A8	N10	T4	E4	H7	PX659522-32
	RVB/Pig-wt/HUN/788-5/2023/G20P[X]	G20	P[X]	I13	R4	C4	M4	A8	N10	T4	E4	H7	PX659533-43
RVC	RVC/Pig-wt/HUN/413-4/2024/G1P[4]	G1	P[4]	I13	R1	C1	M1	A7	N9	T6	E1	H1	PV755149, PX666385-94
	RVC/Pig-wt/HUN/527-7/2024/G6P[X]	G6	P[X]	I13	R1	C1	M1	A7	N9	T1	E1	H1	PV755152, PV755156, PX666395-403
	RVC/Pig-wt/HUN/788-5/2023/G9P[4]	G9	P[4]	I13	R5	C1	M1	A7	N9	T6	E1	H1	PX666404-14
	RVC/Pig-wt/HUN/523-1/2023/G7P[1]	G7	P[1]	I13	R5	C1	M1	A7	N9	T5	E1	H1	PX666415-25

In eight cases the complete genomes were sequenced and classified in accordance with the criteria proposed by the RCWG. The whole genome sequences revealed the backbone constellation of I5-R1-C1-M1-A8-N1-T1/T7-E1/E9-H1 (Table 1.), which corresponds to a Wa-like genomic pattern. Several backbone segments (VP1, VP2, VP3, NSP2 and NSP4) showed high nucleotide identity with both porcine and human Wa-like lineages, indicating shared evolutionary roots. Phylogenetic analyses of all internal segments supported the assigned genotypes and showed clustering with previously described Wa-like porcine RVA strains (Supplementary Figure S2). In two strains, the NSP3 segment belonged to the T7 genotype, which was first detected in a bovine sample [38], but in the recent years has become increasingly prevalent in pigs worldwide and has begun to replace the T1 genotype [27, 39]. The I5 genotype of all VP6 and the A8 genotype of all NSP1 genes are typically associated with porcine origin. Variation was also observed in the NSP4 gene, where one strain (RVA/Pig-wt/HUN/523-11/2023/G2P[34]) carried the E9 genotype, while the remaining strains belonged to genotype E1. Pairwise nucleotide comparisons showed 89.6–90.2% nucleotide identity to previously described E9 reference strains, the closest relative being a Swiss porcine RVA strain collected in 2018.

### Genetic analysis of Hungarian rotavirus B (RVB) strains

**Fig. 3:**
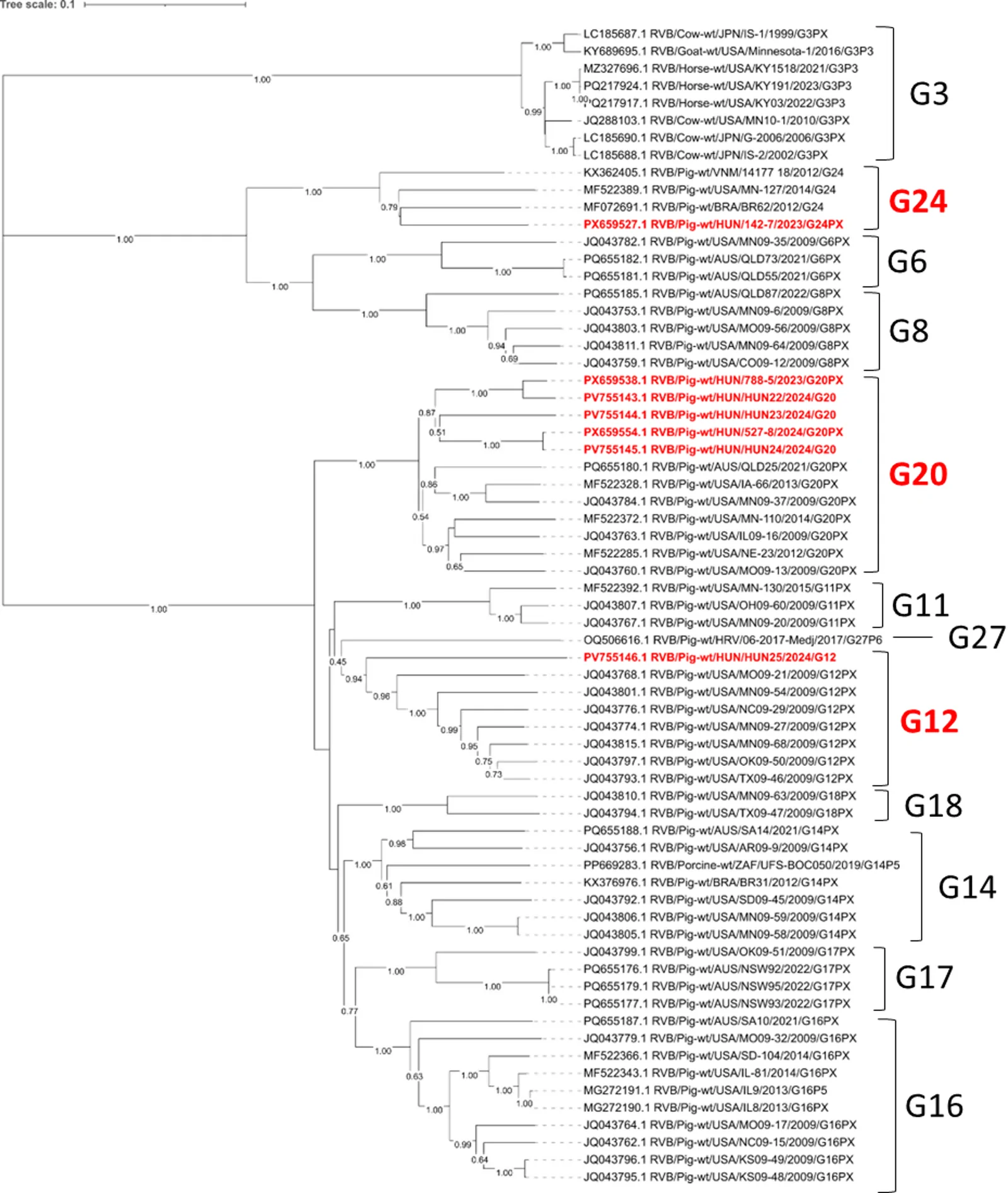
Phylogenetic analysis of the VP7 gene of rotavirus B (RVB). Hungarian RVB sequences are highlighted in red. G-genotypes are indicated on the right side of the tree. Phylogenetic analysis was conducted using MEGA X software with the neighbor-joining method, and node support was assessed using 1000 bootstrap replicates. Reference VP7 sequences were downloaded from GenBank and aligned with the Hungarian sequences to infer genetic relationships

Seven complete VP7 sequences of porcine RVB strains were determined: five of them belonged to the G20 and 1–1 to the G12 and G24 genotypes (Fig. 3.). The G20 sequences shared 86.29% to 99.87% nucleotide identity. Phylogenetic analysis of the VP7 gene showed that the G20 sequences grouped with previously reported porcine RVB strains but formed a separate cluster (bootstrap value: 89%), suggesting local evolution within the Hungarian pig population. The G12 sequence, aligned closely with other porcine G12 strains from the USA, indicating a less divergent lineage. The RVB/Pig-wt/HUN/142-7/2023/G6P[X] strain was classified as genotype G24 and clustered with the limited number of currently available G24 reference sequences. Pairwise nucleotide comparisons showed 81.43–84.65% nucleotide identity to previously reported G24 strains from Brazil, the USA, and Vietnam.

**Fig. 4:**
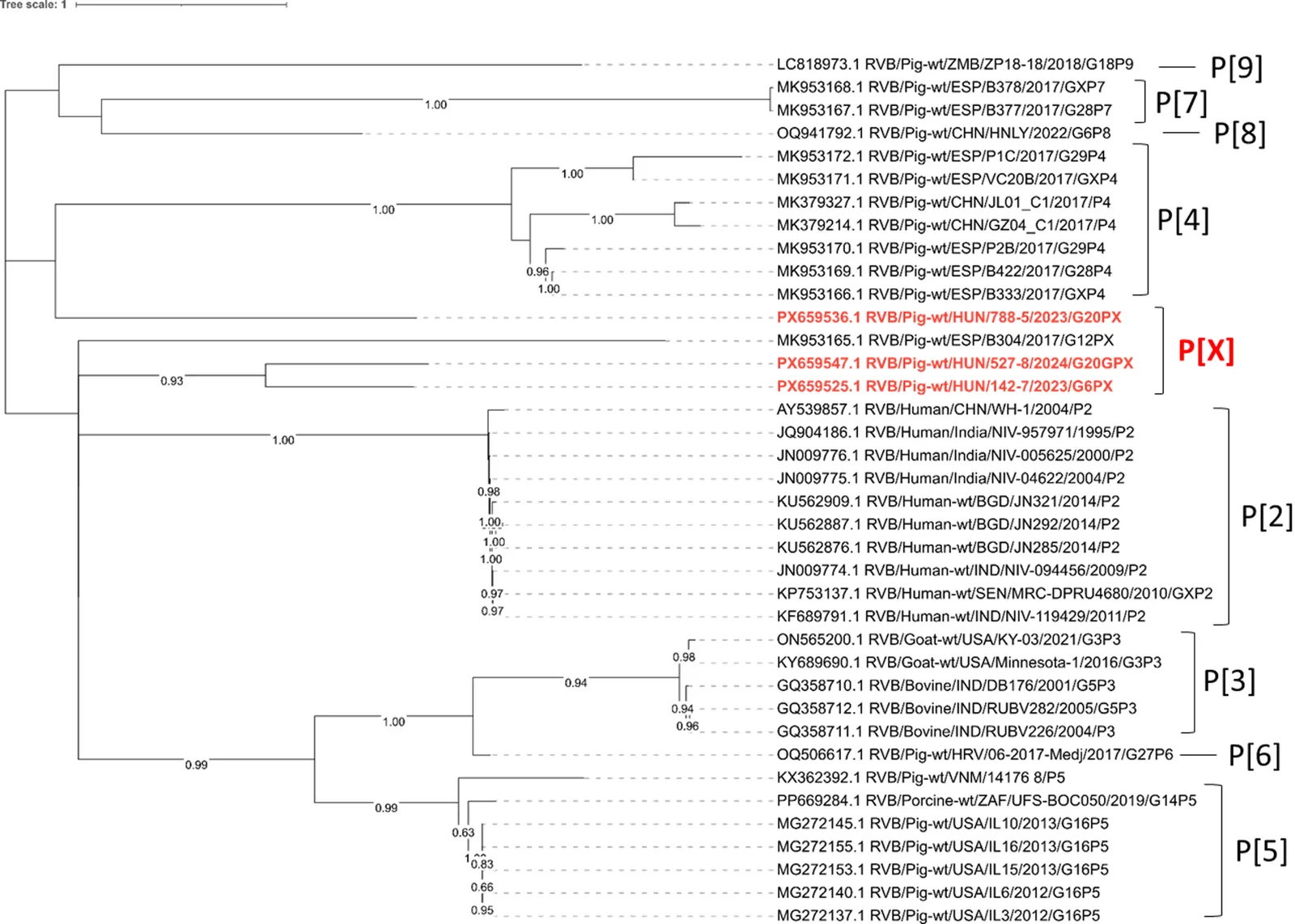
Phylogenetic analysis of the VP4 gene of rotavirus B (RVB). Hungarian RVB sequences are highlighted in red. P-genotypes are indicated on the right side of the tree. Phylogenetic analysis was conducted using MEGA X software with the maximum likelihood method, and node support was assessed using 1000 bootstrap replicates. Reference VP4 sequences were downloaded from GenBank and aligned with the Hungarian sequences to infer genetic relationships

Three complete VP4 sequences were determined, but none of them could be classified into any previously described genotypes, therefore these are designated as P[X] (Fig. 4.). In the phylogenetic tree, the Hungarian strains clustered away from all reference sequences. Pairwise nucleotide distance analysis further supported this observation (Supplementary Table S2)*.* One Hungarian VP4 sequence (PX659536) showed relatively high nucleotide identity (89.07%) to a partial Russian RVB VP4 sequence, which likewise could not be assigned to any established P genotype by the authors [29]. The remaining two Hungarian VP4 sequences showed the highest nucleotide similarity to partial Chinese and Vietnamese sequences; however, nucleotide identity within the overlapping regions ranged from 75.58% to 79.6%, which is below the currently proposed genotype demarcation threshold. Given the currently limited reference data available for RVB, the Hungarian strains cannot be assigned to any known genotype using the suggested 80% cut-off value.

Whole-genome sequencing of these strains revealed the following backbone constellations: I11/I13-R4-C4-M4-A8-N10-T4-E4-H7 (Table 1.). With the exception of VP6, all internal gene segments were conserved across the analyzed strains. This backbone configuration highly resembles those described in North American RVB strains, which often share conserved internal gene segments, such as VP1, VP2, VP3, NSP3, NSP4 and NSP5. Based on nBLAST analysis and pairwise sequence comparisons, the NSP genes, particularly the NSP1, NSP2 and NSP3, also showed high identity with porcine associated genogroups. Phylogenetic analyses of all genome segments supported the assigned genotypes and showed clustering with previously described porcine RVB strains, with no evidence of recent interspecies reassortment (Supplementary Figure S3). The only variation among the Hungarian strains was observed in the VP6 gene, where both I11 and I13 genotypes were detected.

### Genetic analysis of Hungarian rotavirus C (RVC) strains

**Fig. 5:**
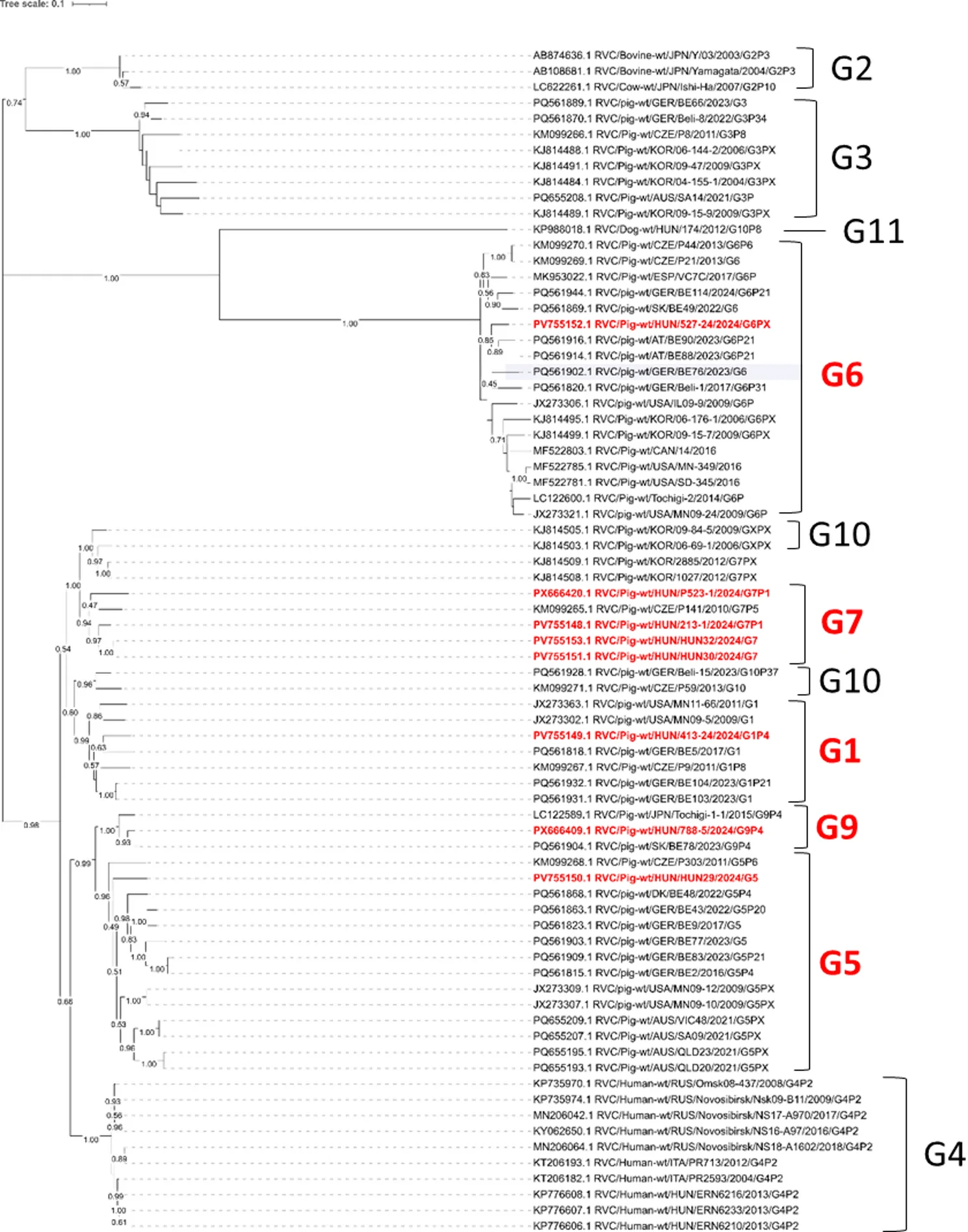
Phylogenetic analysis of the VP7 gene of rotavirus C (RVC). Hungarian RVC sequences are highlighted in red. G-genotypes are indicated on the right side of the tree. Phylogenetic analysis was conducted using MEGA X software with the maximum likelihood method. Node support was assessed using 1000 bootstrap replicates. Reference VP7 sequences were downloaded from GenBank and aligned with the Hungarian sequences to infer genetic relationships

Eight complete VP7 gene sequences of RVC were obtained and classified into five G genotypes: G7 (*n =* 4), G1 (*n =* 1), G5 (*n =* 1), G6 (*n =* 1) and G9 (*n =* 1) (Fig. 5.). The G7 sequences exhibited 85.09–99.8% nucleotide identity, forming a monophyletic group. Phylogenetic analysis showed that the Hungarian RVC strains of the different G genotypes clustered with previously described porcine RVC strains from Europe, including sequences from Germany, Belgium, the Czech Republic, Austria, and Poland.

**Fig. 6:**
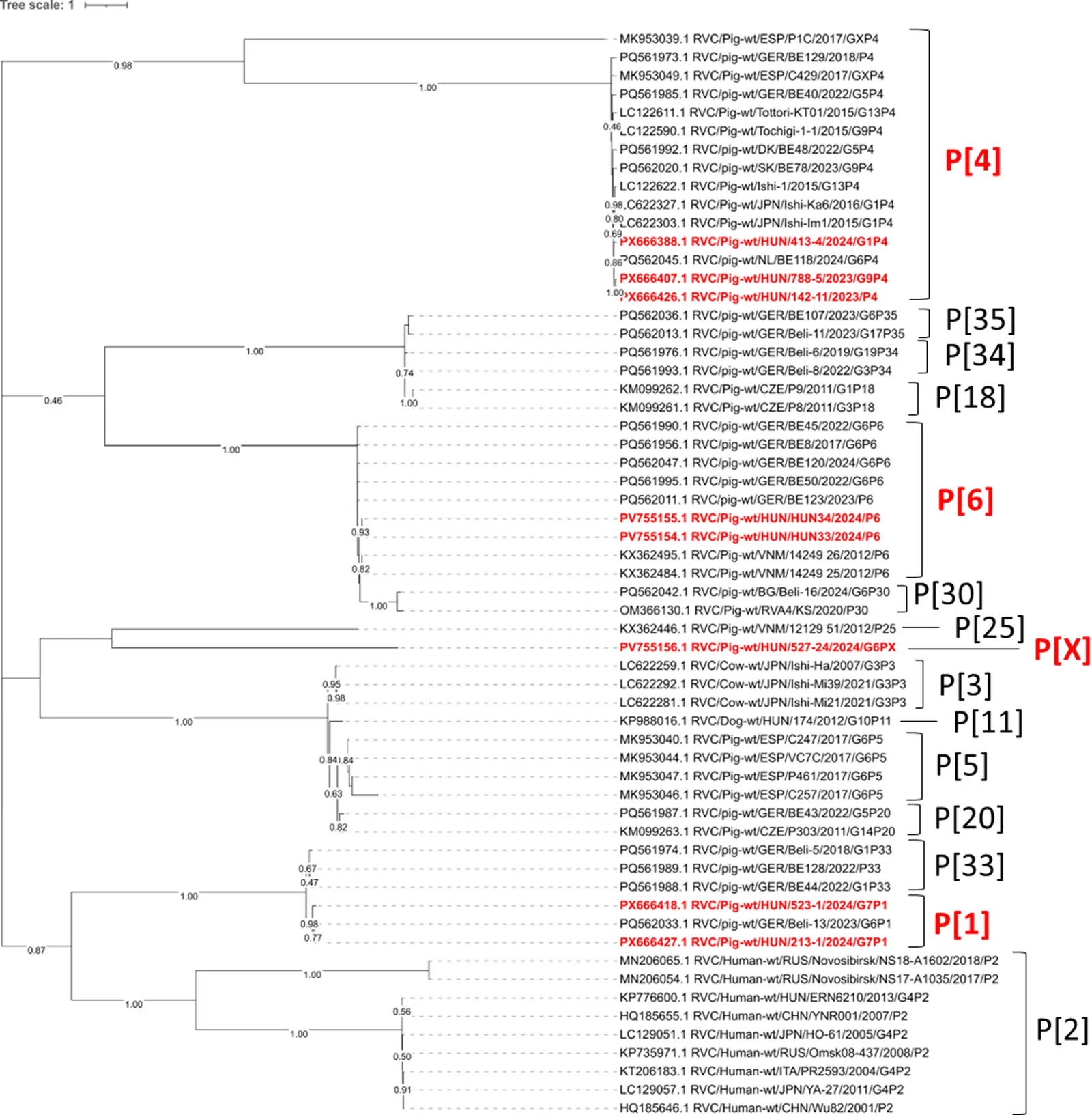
Phylogenetic analysis of the VP4 gene of rotavirus C (RVC). Hungarian RVC sequences are highlighted in red. P-genotypes are indicated on the right side of the tree. Phylogenetic analysis was conducted using MEGA X software with the maximum likelihood method. Node support was assessed using 1000 bootstrap replicates. Reference VP4 sequences were downloaded from GenBank and aligned with the Hungarian sequences to infer genetic relationships

VP4 genotyping identified three P-genotypes: P[6] (*n =* 2), P[1] (*n =* 2), P[4] (*n =* 3) (Fig. 6.). One of the VP4 gene sequences (designated P[X]) showed only 79.50–85.28% nucleotide identity with reference porcine RVC VP4 sequences available in GenBank (Supplementary Table S3). The most similar reference sequences included porcine strains from Germany, the Czech Republic, and Vietnam. Although the observed nucleotide identities were close to the proposed genotype cut-off threshold, phylogenetic analysis demonstrated that the Hungarian strain formed a distinct lineage and did not cluster within any established P genotype. Together, these findings suggest that the Hungarian P[X] sequence may represent a novel VP4 genotype. Phylogenetic analysis supported its distant clustering from established P genotypes. G/P genotype combinations detected included G7P[1] (*n =* 2), G7P[6] (*n =* 1), G1P[4] (*n =* 1), G9P[4] (*n =* 1) and G6P[X] (*n =* 1).

The whole-genome constellations of the four Hungarian strains were slightly different, but all of them had a Cowden-like porcine backbone, with several internal genes belonging to genotype 1 (Table 1.). This Cowden-like constellation is one of the most common genomic patterns in swine RVC. Conserved internal gene genotypes included C1 (VP2), M1 (VP3), I13(VP6), E1 (NSP4), and H1 (NSP5), which were identical across all analyzed strains. Variation among the genome constellations was mainly observed in the outer capsid VP4 and VP7 genes, as well as in VP1 and NSP3. Two VP1 genotypes (R1 and R5) and three NSP3 genotypes (T1, T5, and T6) were identified among the Hungarian strains. Phylogenetic analyses of all genome segments supported the assigned genotypes and showed clustering with previously described porcine RVC strains, with no evidence of recent interspecies reassortment (Supplementary Figure S3).

## Discussion

RVs are among the most important viral pathogens associated with diarrhea in suckling and weaned piglets. Although RVA is a well-characterized cause of piglet diarrhea, the significance of other RV species remains less defined. Data on the prevalence and genetic diversity of circulating RV strains in Hungarian pig herds are still very limited. A prevalence study conducted in Hungary between 2016 and 2018 detected RVA and RVC on 11 out of 17 farms, with an overall positivity rate of 21% of samples from diarrheic and healthy pigs combined, though detection was significantly higher in the clinically ill animals [40]. In this present study, G/P genotyping of Hungarian RV strains was performed from samples of diarrheic piglets. Altogether, 80.5% of the tested samples were positive for at least one RV, with RVA and RVC being equally prevalent (54.5%), while RVB was less frequently detected (40.3%). However, the observed detection rates are influenced by the sampling strategy, as a proportion of samples were pre-selected based on RVA positivity. It is important to note that coinfections involving multiple RVs and other enteric pathogens were common, so the clinical symptoms of the examined piglets were not necessarily caused solely by RV infection. The presence of additional pathogens may have contributed to or exacerbated disease severity, and in some cases, these may have even acted as primary causative agents. Several emerging and also some well-known enteric pathogens were identified in a subset of the examined samples, such as *Escherichia* spp., astrovirus, kobuvirus, *Enterococcus* spp., sapovirus, and enterovirus G. However, the detailed metagenome analysis of the sampled piglets falls outside the scope of this work and represents a limitation of the present study. Nevertheless, the successful recovery of complete RVA, RVB, and RVC genome sequences using the PathoSense BV metagenomic Nanopore workflow highlights the versatility of this approach. Similar applications of the same workflow have previously enabled whole-genome characterization of porcine RVs and other enteric viruses from clinical samples, while simultaneously providing information on co-circulating pathogens [29, 41].

The detected RVA strains were classified into multiple G/P genotype combinations characteristic of porcine RVA. For example, G4, G5 and G9 genotypes were identified in the samples, paired with P[7], P[13], and P[23]. In the last decade, the G5P[7] genotype combination has been reported as the most widespread among porcine RVA strains globally [42]. However, a shifting trend, towards the emerging G9P[23] was reported in European countries, such as Croatia [43], Germany [44] and Spain [45] and our results also identified G9P[23] as the most frequent genotype combination among detected Hungarian strains.

Phylogenetic analysis of the Hungarian RVA VP7/VP4 sequences revealed that they cluster mostly with porcine RVA strains from Europe and Asia rather than with human RVA lineages. The only exceptions were the G4 and P[6] genotypes, which closely resembled porcine-like G4P[6] RVA strains previously detected in human patients from Hungary more than a decade ago, suggesting zoonotic transmission from pigs [37]. Interestingly, a closely related G4P[6] porcine strain was reported in Slovakia in 2017, likely originating from Hungary, further supporting cross-border circulation of zoonotic RVA strains in the region [46]. The genome constellation of the Hungarian strains revealed a Wa-like backbone across most of their internal segments. While this genomic pattern was initially described in association with human strains, it has since been widely documented in porcine RVA as well [13, 47, 48], which likely reflects either ancient reassortment events or a shared evolutionary origin between human and porcine Wa-like lineages. The detection of the relatively uncommon E9 NSP4 genotype in the G2P[34] strain further expands the known geographic distribution of this genotype. The Hungarian E9 sequence showed the highest nucleotide identity to previously reported E9 strains from Switzerland, Belgium, and South Africa, indicating that genetically related E9 variants circulate in geographically distant swine populations [47, 49].

RVB was also detected in several diarrheic piglets, which is noteworthy since RVB is less commonly targeted in diagnostic screening compared to RVA. Although its clinical relevance in pigs is still not fully understood, RVB has been increasingly reported in diarrhea outbreak cases in pigs worldwide [24, 50]. Phylogenetic studies have shown that porcine RVB strains are highly diverse, especially in the VP7 gene, as according to Shepherd et al. [10] at least 26 different G genotypes can be identified based on an 80% nucleotide identity cut-off value. With the increasing application of NGS technologies, the number of newly recognized RVB genotypes continues to grow rapidly, highlighting the complexity of this virus. In this study seven complete VP7 sequences were obtained, and classified into the G12, G20 and G24 genotypes. The G20 strains formed a distinct cluster, suggesting local evolution in the Hungarian pig population, while the G12 strain showed close similarity to North American reference sequences. One Hungarian strain belonged to the relatively rarely reported G24 genotype, which was first described in 2018 from Brazilian and Vietnamese strains [51]. Its detection in Hungary provides additional evidence for the circulation of this genotype in Europe. Three full-length VP4 sequences were determined, but it could not be assigned to any currently recognized P genotypes. To date, nine porcine RVB VP4 genotypes have been described, including the recently identified P[9] from Zambia, which similarly to our Hungarian strains, showed considerable genetic divergence from previously known sequences [52]. All other successfully sequenced genome segments corresponded to established porcine genotypes, and no indication of reassortment involving non-porcine strains has been found. This is consistent with current knowledge on RVB evolution, which (unlike RVA) has not shown evidence of cross-species reassortment events between humans and domestic animals [10].

RVC has become increasingly recognized as an important cause of piglet diarrhea worldwide, in some cases rivaling RVA in prevalence. Reports from Italy [19] and Asia [30, 53] have shown its major role in diarrhea outbreaks among neonatal and weaned pigs, underscoring its clinical relevance. Our detection of RVC in more than half of the diarrheic piglets in Hungary aligns with these global observations and expands the known geographic range of the virus. The high genetic diversity of porcine RVC, greater than that of human or bovine strains [54], likely reflects its long-term circulation and large virus populations in swine, with pigs serving as the main reservoir of RVC genetic diversity and a potential source of zoonotic transmission. Notably, an Indian study reported porcine RVCs carrying VP6 genes of human origin (I2 genotype), providing further evidence for possible interspecies transmission [55].

Phylogenetic analyses and genotyping of the Hungarian strains revealed five distinct G genotypes (G1, G5, G6, G7 and G9). The G7 sequences formed a well-supported monophyletic cluster with other European porcine RVC strains. These strains showed high sequence identity with porcine reference sequences from Germany, Belgium, Austria, and the Czech Republic indicating circulation of closely related lineages in Central Europe. VP4 genotyping identified three established genotypes, but one sequence showed less than 85% identity to known porcine RVC VP4 genes, suggesting the presence of a novel P genotype. Phylogenetic analysis supported the distant placement of the Hungarian P[X] sequence from known types, highlighting the ongoing evolution of RVC in swine where 23 different P genotypes have already been described [56]. The complete genome constellation of all analyzed strains revealed Cowden-like (G1) genomic constellations, which is among the most common full-genome configurations in pigs [30].

The limitation of our study is that the sampling strategy may have introduced bias, as the samples were partly pre-selected based on RVA positivity. In addition, the lack of standardized classification criteria for RVB and RVC limits the formal assignment of genetically distinct strains, including those identified as putative novel genotypes.

## Conclusion

In summary, this study provides a comprehensive molecular characterization of RVA, RVB, and RVC strains circulating in diarrheic piglets. A high prevalence of RV infections and frequent coinfections were observed, highlighting the complexity of enteric infections in piglets. Complete genome analysis revealed Wa-like genomic constellations associated with previously described porcine G/P genotype combinations in RVA strains. RVB and RVC analyses identified genetically distinct strains, including putative novel VP4 genotypes. Our phylogenetic analyses provide valuable insights into the local genetic diversity and evolutionary dynamics of porcine RVs and may contribute to the development of improved classification systems for RVB and RVC. These findings also highlight the importance of integrated surveillance strategies that account for multiple RV species and coinfecting pathogens.

## Methods

### Sample collection

A total of 77 fecal swab samples, representing 202 diarrheic piglets originating from 19 farrow-to-finish swine farms across Hungary, were analyzed, of which 33 were obtained from the Livestock Diagnostic Center of the University of Veterinary Medicine. The dataset included both individual and pooled samples, with pooled samples representing between 2 and 10 animals from the same farm and age group. Detailed information regarding farm locations, sampling dates, age categories, and the number of animals represented by each sample is provided in Supplementary Table S1. The swabs were suspended in phosphate-buffered saline (PBS), thoroughly vortexed, centrifuged (300 × g for 5 min) and filtered to remove solid debris. Viral enrichment was subsequently performed by Benzonase Nuclease (Millipore, Burlington, MA, USA) treatment for 1 h at 37 °C to reduce host nucleic acids. Before further processing, the suspensions were stored at 4 °C.

### Rotavirus detection by RT-qPCR

Nucleic acid extraction from the fecal swab samples were performed using the Quick-DNA/RNA Viral Kit (Zymo Research, Tustin, CA, USA). Then RT-qPCR was used to detect viral RNA in the samples. The RT-qPCR assays were run on a Q qPCR machine (Quantabio, Beverly, MA, USA). For RV detection, previously described specific primers and probes were used (Table 2).Each reaction contained 10 µlqScript^tm^XLT One-Step RT-qPCR ToughMix (Quantabio), 2 µl extracted RNA, 900 nM of each primer and 250 nM of each probe in 20 µl final volume. RVB and RVC assays were run in duplex format in a single reaction tube. The thermal cycling conditions were as follows: initial denaturation at 95 °C for 3 min, followed by 40 cycles of 95 °C for 10 s and 60 °C for 30 s. Samples with cycle threshold (Ct) values higher than 36 were considered negative.

**Table 2:** Primer and probe sets used for the detection of rotavirus A (RVA), rotavirus B (RVB), and rotavirus C (RVC) by RT-qPCR

	Primer	Primer sequence	Target gene	Position (bp)	Ref. sequence	Reference
RVA	RVA7-1 F	5’-rcatracccyctatgagcac-3’	NSP3	997–1016	ON563327.1	[57]
	Rota NVP3-R	5’-ggtcacataacgcccc-3’		1059–1074		[28]
	RVA7probe1	5’-FAM-atagttaaaagctaacactgtcaaaaacctaaa-BHQ-3’		1018–1050		[28]
RVB	RVB_VP6_for	5’-trtggkgwcaraaratagcrat-3’	VP6	1155–1175	KR052718.1	[19]
	RVB_VP6_rev	5’-acctytcgaagcactyccwtt-3’		1223–1243		[19]
	RVB_probe	5’-TxRed-tgatccggcgtcrgct-BHQ-3’		1204–1219		[19]
RVC	RVC6-3 F	5’-gttgcatccgtgaagagaatg-3’	VP6	1189–1209	AB889516.1	[28]
	C4	5’-agccacatagttcacatttcatcc-3’		1338–1361		[28]
	RVC_probe	5’-HEX-accatgtagcatgattcacgaatgggt-BHQ-3’		1258–1284		[28]

### Nanopore sequencing and genome assembly

Nanopore metagenomic sequencing was conducted following the workflow developed by PathoSense BV (Merelbeke, Belgium) [41]. Firstly, reverse transcription from the extracted RNA samples were performed using the SuperScript IV Reverse Transcriptase (ThermoFisher Scientific, Waltham, MA, USA) with random hexamer primers. It was followed by PCR amplification of the generated cDNA using the KAPA HiFi HotStart ReadyMix (Roche, Basel, Switzerland). To purify the amplified DNA CleanNGS magnetic beads (CleanNA, Waddinxveen, The Netherlands) were used at a 1:1 ratio. The concentration and quality of the purified DNA were assessed using a NanoDrop™ One/OneC Microvolume UV-Vis Spectrophotometer (Thermo Scientific™, USA). Sequencing libraries were prepared with the SQK-RBK004 Rapid Barcoding Kit (Oxford Nanopore Technologies, Oxford, UK), and before adapter ligation the barcoded library was purified using CleanNGS magnetic beads once again. Sequencing was performed using the MinION device with flow cells version R9 or R10 (Oxford Nanopore Technologies) for 12 h. Fast5 files were generated using MinKNOW software (v.24.11.10). Subsequent basecalling, demultiplexing, and quality filtering were performed using Dorado (v.0.7.9) in super-accurate mode, using the model appropriate for the flow cell chemistry applied in each sequencing run. Reads with a Q-score below 7 or shorter than 200 bp in length were excluded during basecalling. The mean Q-score of the raw reads was calculated using the Datamash tool implemented in the Galaxy Project online platform [58]. For downstream analysis, Geneious Prime software (Biomatters Ltd., Auckland, New Zealand) was used. Filtered reads were taxonomically classified using BLASTn and BLASTx against a customized viral database containing all publicly available RVA, RVB and RVC sequences from GenBank (accessed June 2025). Reads showing significant similarity to RVs were retained for further analysis. Genome reconstruction was performed using both de novo assembly and reference-guided mapping approaches. De novo assembly was carried out using Flye (v.2.9.1) [59], and the resulting contigs were aligned to RV reference genomes using Minimap2 (v.2.24) [60] to verify segment identity. In parallel, reads were mapped to reference sequences using Minimap2 (v.2.24), and consensus sequences were generated from mappings supported by a minimum of 250 reads and high sequence coverage. Assembly polishing was performed within the Galaxy Project platform using the integrated Medaka pipeline. All sequence alignments and assemblies were manually inspected and curated to ensure accuracy.

### Genotyping and phylogenetic analysis

Nucleotide sequences of all 11 segments (VP1–4, VP6, VP7 and NSP1–5) from RVA, RVB, and RVC were aligned using the E-INS-i algorithm in MAFFT v7 [61]. For RVA, VP4 and VP7 phylogenetic trees were constructed using the neighbor-joining method with the p-distance model. For RVB, phylogenetic trees were constructed using the maximum likelihood (VP4) and neighbor-joining (VP7) method with the General Time Reversible (GTR) and the p-distance model, respectively. The RVC VP4 and VP7 sequences were analyzed using the maximum likelihood method with the General Time Reversible (GTR) model. For all RVA, RVB and RVC internal genome segments, phylogenetic trees were generated using the neighbor-joining method with the p-distance model. Rates among sites were modeled using a gamma distribution with invariant sites (G + I) in all cases. Different phylogenetic methods were applied depending on the gene and dataset to ensure appropriate resolution of intra-genotypic relationships [62]. All phylogenetic trees were generated in MEGA X with 1000 bootstrap replicates to assess the robustness of the branches. Reference sequences used for the phylogenetic comparisons were selected to include at least two representative strains of each relevant genotype. The resulting phylogenetic trees were visualized and annotated using the Interactive Tree Of Life (iTOL) v6 [63].

For RVA, the initial genotyping of all 11 segments were performed using the Subspecies Classification Service of the BV-BRC platform (v.3.53.3) [64], which is recommended by the Rotavirus Classification Working Group (RCWG). It utilizes the pplacer tool for phylogenetic placement against curated reference datasets maintained according to ICTV standards. For RVB and RVC, preliminary genotypes of the internal segments were assigned using BLASTn searches against the National Center for Biotechnology Information (NCBI) database. Previously published nucleotide identity cutoffs were applied: 76–83% for RVB [10] and an 85% cutoff for RVC in line with the VP4 and VP7 recommendation, due to the absence of established thresholds for its other segments [56]. These genotype assignments were subsequently verified and supported by phylogenetic analyses of all genome segments.

The RVA, RVB and RVC sequences identified in this study have been uploaded in the NCBI GenBank under the accession numbers: PV755122–PV755156, PX659522–PX659554, PX666385–PX666427 and PX677582–PX677669.

## Supplementary Information


Supplementary Material 1. Supplementary Figure 1: Distribution of RVA, RVB, and RVC positive samples and coinfections. The Venn diagram was generated using the InteractiVenn online tool [65]. Supplementary Figure 2: Phylogenetic analysis of RVA VP1, VP2, VP3, VP6, NSP1, NSP2, NSP3, NSP4 and NSP5 genes. Phylogenetic trees were constructed using the neighbor-joining method with 1000 bootstrap replicates. Hungarian strains identified in this study are highlighted in red. Supplementary Figure 3: Phylogenetic analysis of RVB VP1, VP2, VP3, VP6, NSP1, NSP2, NSP3, NSP4 and NSP5 genes. Phylogenetic trees were constructed using the neighbor-joining method with 1000 bootstrap replicates. Hungarian strains identified in this study are highlighted in red. Supplementary Figure 4: Phylogenetic analysis of RVC VP1, VP2, VP3, VP6, NSP1, NSP2, NSP3, NSP4 and NSP5 genes. Phylogenetic trees were constructed using the neighbor-joining method with 1000 bootstrap replicates. Hungarian strains identified in this study are highlighted in red [11]. 



Supplementary Material 2. Supplementary Table 1: Detailed information on sample collection and the swine farms included in this study.Supplementary Table 2: Pairwise nucleotide identities (%) between the putative novel RVB VP4 genotypes and representative reference strains. Values were calculated from the evolutionary divergence matrix generated in MEGAX. Supplementary Table 3: Pairwise nucleotide identities (%) between the detected RVC VP4 strains and representative reference strains. Values were calculated from the evolutionary divergence matrix generated in MEGAX.


## Data Availability

The genome sequences have been deposited in the NCBI GenBank under the accession numbers: PV755122–PV755156, PX659522–PX659554, PX666385–PX666427 and PX677582–PX677669.

## References

[1] Matthijnssens J, Attoui H, Bányai K, Brussaard CPD, Danthi P, del Vas M, et al. ICTV Virus Taxonomy Profile: Sedoreoviridae 2022. J Gen Virol. 2022;103:001782. 10.1099/jgv.0.001782. 36215107 PMC12643109

[2] Desselberger U, Rotaviruses. Virus Res. 2014;190:75–96. 10.1016/j.virusres.2014.06.016. 25016036

[3] Flewett TH, Woode GN. The rotaviruses. Arch Virol. 1978;57:1–23. 10.1007/BF01315633. 77663 PMC7087197

[4] Matthijnssens J, Ciarlet M, McDonald SM, Attoui H, Bányai K, Brister JR, et al. Uniformity of rotavirus strain nomenclature proposed by the Rotavirus Classification Working Group (RCWG). Arch Virol. 2011;156:1397–413. 10.1007/s00705-011-1006-z. 21597953 PMC3398998

[5] Martella V, Bányai K, Matthijnssens J, Buonavoglia C, Ciarlet M. Zoonotic aspects of rotaviruses. Vet Microbiol. 2010;140:246–55. 10.1016/j.vetmic.2009.08.028. 19781872

[6] Matthijnssens J, Desselberger U. Genome Diversity and Evolution of Rotaviruses. Genome Plasticity and Infectious Diseases. John Wiley & Sons, Ltd; 2012. p. 214–41. 10.1128/9781555817213.ch13.

[7] Rotavirus Classification Working Group: RCWG, Rega Institute KU, Leuven. https://rega.kuleuven.be/cev/viralmetagenomics/virus-classification/rcwg.Accessed 11 Aug. 2025.

[8] Vlasova AN, Amimo JO, Raev SA, Saif LJ. Rotaviruses and Reoviruses. Diseases of Swine. John Wiley & Sons, Ltd; 2025. p. 797–812. 10.1002/9781394179466.ch38.

[9] Matthijnssens J, Ciarlet M, Rahman M, Attoui H, Bányai K, Estes MK, et al. Recommendations for the classification of group A rotaviruses using all 11 genomic RNA segments. Arch Virol. 2008;153:1621–9. 10.1007/s00705-008-0155-1. 18604469 PMC2556306

[10] Shepherd F, Herrera-Ibata D, Porter E, Homwong N, Hesse R, Bai J, et al. Whole Genome Classification and Phylogenetic Analyses of Rotavirus B strains from the United States. Pathogens. 2018;7:44. 10.3390/pathogens7020044. 29670022 PMC6027208

[11] Suzuki T, Hasebe A. A provisional complete genome-based genotyping system for rotavirus species C from terrestrial mammals. J Gen Virol. 2017;98:2647–62. 10.1099/jgv.0.000953. 29039732

[12] Matthijnssens J, Ciarlet M, Heiman E, Arijs I, Delbeke T, McDonald SM, et al. Full Genome-Based Classification of Rotaviruses Reveals a Common Origin between Human Wa-Like and Porcine Rotavirus Strains and Human DS-1-Like and Bovine Rotavirus Strains. J Virol. 2008;82:3204–19. 10.1128/jvi.02257-07. 18216098 PMC2268446

[13] Matthijnssens J, Van Ranst M. Genotype constellation and evolution of group A rotaviruses infecting humans. Curr Opin Virol. 2012;2:426–33. 10.1016/j.coviro.2012.04.007. 22683209

[14] Dhama K, Chauhan RS, Mahendran M, Malik SVS. Rotavirus diarrhea in bovines and other domestic animals. Vet Res Commun. 2009;33:1–23. 10.1007/s11259-008-9070-x. 18622713 PMC7088678

[15] Sun M, Li T, Liu W, Guo Z, Wang Z, Jiang J, et al. Severe Diarrhea Outbreaks in Newborn Piglets in China Associated With Porcine Rotavirus B. Transbound Emerg Dis. 2025;2025:5588912. 10.1155/tbed/5588912. 41323144 PMC12662673

[16] Vlasova A, Amimo J, Saif L. Porcine Rotaviruses: Epidemiology, Immune Responses and Control Strategies. Viruses. 2017;9:48. 10.3390/v9030048. 28335454 PMC5371803

[17] Mebus C, Underdahl N, Rhodes M, Twiehaus M. Calf Diarrhea (Scours): Reproduced with a Virus from a Field Outbreak. Nebraska Agricultural Experiment Station: Historical Research Bulletins; 1969.

[18] Bishop RF, Davidson GP, Holmes IH, Ruck BJ, VIRUS PARTICLES IN EPITHELIAL CELLS OF DUODENAL MUCOSA FROM CHILDREN WITH ACUTE NON-BACTERIAL GASTROENTERITIS. Lancet. 1973;302:1281–3. 10.1016/S0140-6736(73)92867-5. 4127639

[19] Ferrari E, Salogni C, Martella V, Alborali GL, Scaburri A, Boniotti MB. Assessing the Epidemiology of Rotavirus A, B, C and H in Diarrheic Pigs of Different Ages in Northern Italy. Pathogens. 2022;11:467. 10.3390/pathogens11040467. 35456143 PMC9025647

[20] Amimo JO, Junga JO, Ogara WO, Vlasova AN, Njahira MN, Maina S, et al. Detection and genetic characterization of porcine group A rotaviruses in asymptomatic pigs in smallholder farms in East Africa: Predominance of P[8] genotype resembling human strains. Vet Microbiol. 2015;175:195–210. 10.1016/j.vetmic.2014.11.027. 25541378

[21] Theil KW, Saif LJ, Moorhead PD, Whitmoyer RE. Porcine rotavirus-like virus (group B rotavirus): characterization and pathogenicity for gnotobiotic pigs. J Clin Microbiol. 1985;21:340–5. 10.1128/jcm.21.3.340-345.1985. 2984243 PMC271660

[22] Chang KO, Parwani AV, Smith D, Saif LJ. Detection of group B rotaviruses in fecal samples from diarrheic calves and adult cows and characterization of their VP7 genes. J Clin Microbiol. 1997;35:2107–10. 10.1128/jcm.35.8.2107-2110.1997. 9230391 PMC229912

[23] Uprety T, Sreenivasan CC, Hause BM, Li G, Odemuyiwa SO, Locke S, et al. Identification of a Ruminant Origin Group B Rotavirus Associated with Diarrhea Outbreaks in Foals. Viruses. 2021;13:1330. 10.3390/v13071330. 34372536 PMC8310321

[24] Li Q, Wang Z, Jiang J, He B, He S, Tu C, et al. Outbreak of piglet diarrhea associated with a new reassortant porcine rotavirus B. Vet Microbiol. 2024;288:109947. 10.1016/j.vetmic.2023.109947. 38101077

[25] Molinari BLD, Possatti F, Lorenzetti E, Alfieri AF, Alfieri AA. Unusual outbreak of post-weaning porcine diarrhea caused by single and mixed infections of rotavirus groups A, B, C, and H. Vet Microbiol. 2016;193:125–32. 10.1016/j.vetmic.2016.08.014. 27599939

[26] Marthaler D, Homwong N, Rossow K, Culhane M, Goyal S, Collins J, et al. Rapid detection and high occurrence of porcine rotavirus A, B, and C by RT-qPCR in diagnostic samples. J Virol Methods. 2014;209:30–4. 10.1016/j.jviromet.2014.08.018. 25194889

[27] Vidal A, Martín-Valls GE, Tello M, Mateu E, Martín M, Darwich L. Prevalence of enteric pathogens in diarrheic and non-diarrheic samples from pig farms with neonatal diarrhea in the North East of Spain. Vet Microbiol. 2019;237:108419. 10.1016/j.vetmic.2019.108419. 31585655 PMC7117353

[28] Otto PH, Rosenhain S, Elschner MC, Hotzel H, Machnowska P, Trojnar E, et al. Detection of rotavirus species A, B and C in domestic mammalian animals with diarrhoea and genotyping of bovine species A rotavirus strains. Vet Microbiol. 2015;179:168–76. 10.1016/j.vetmic.2015.07.021. 26223422

[29] Krasnikov N, Gulyukin A, Aliper T, Yuzhakov A. Complete genome characterization by nanopore sequencing of rotaviruses A, B, and C circulating on large-scale pig farms in Russia. Virol J. 2024;21:289. 10.1186/s12985-024-02567-9. 39538316 PMC11562526

[30] Saif LJ, Bohl EH, Theil KW, Cross RF, House JA. Rotavirus-like, calicivirus-like, and 23-nm virus-like particles associated with diarrhea in young pigs. J Clin Microbiol. 1980;12:105–11. 10.1128/jcm.12.1.105-111.1980. 6252238 PMC273530

[31] Bridger JC, Pedley S, McCrae MA. Group C rotaviruses in humans. J Clin Microbiol. 1986;23:760–3. 10.1128/jcm.23.4.760-763.1986. 3009541 PMC362832

[32] Chang KO, Nielsen PR, Ward LA, Saif LJ. Dual infection of gnotobiotic calves with bovine strains of group A and porcine-like group C rotaviruses influences pathogenesis of the group C rotavirus. J Virol. 1999;73:9284–93. 10.1128/JVI.73.11.9284-9293.1999. 10516037 PMC112963

[33] Marton S, Mihalov-Kovács E, Dóró R, Csata T, Fehér E, Oldal M, et al. Canine rotavirus C strain detected in Hungary shows marked genotype diversity. J Gen Virol. 2015;96:3059–71. 10.1099/jgv.0.000237. 26297005

[34] Bányai K, Jiang B, Bogdán Á, Horváth B, Jakab F, Meleg E, et al. Prevalence and molecular characterization of human group C rotaviruses in Hungary. J Clin Virol. 2006;37:317–22. 10.1016/j.jcv.2006.08.017. 16996791

[35] Marthaler D, Rossow K, Culhane M, Collins J, Goyal S, Ciarlet M, et al. Identification, phylogenetic analysis and classification of porcine group C rotavirus VP7 sequences from the United States and Canada. Virology. 2013;446:189–98. 10.1016/j.virol.2013.08.001. 24074581

[36] Wang Y, Porter EP, Lu N, Zhu C, Noll LW, Hamill V, et al Whole-genome classification of rotavirus C and genetic diversity of porcine strains in the USA. J Gen Virol. 2021;102. 10.1099/jgv.0.001598. 33950806

[37] Papp H, Borzák R, Farkas S, Kisfali P, Lengyel G, Molnár P, et al. Zoonotic transmission of reassortant porcine G4P[6] rotaviruses in Hungarian pediatric patients identified sporadically over a 15 year period. Infect Genet Evol. 2013;19:71–80. 10.1016/j.meegid.2013.06.013. 23792183

[38] Full genomic analysis of. a porcine–bovine reassortant G4P[6] rotavirus strain R479 isolated from an infant in China Wang .Wiley Online Library. Journal of Medical Virology. 2010. https://onlinelibrary.wiley.com/doi/10.1002/jmv.21760. Accessed 15 Aug 2025. 10.1002/jmv.21760 20419827

[39] Strydom A, Segone N, Coertze R, Barron N, Strydom M, O’Neill HG. Phylogenetic Analyses of Rotavirus A, B and C Detected on a Porcine Farm in South Africa. Viruses. 2024;16:934. 10.3390/v16060934. 38932226 PMC11209240

[40] Valkó A, Marosi A, Cságola A, Farkas R, Rónai Z, Dán Á. Frequency of diarrhoea-associated viruses in swine of various ages in Hungary. Acta veterinaria Hungarica. 2019;67:140–50. 10.1556/004.2019.016. 30922088

[41] Theuns S, Vanmechelen B, Bernaert Q, Deboutte W, Vandenhole M, Beller L, et al. Nanopore sequencing as a revolutionary diagnostic tool for porcine viral enteric disease complexes identifies porcine kobuvirus as an important enteric virus. Sci Rep. 2018;8:9830. 10.1038/s41598-018-28180-9. 29959349 PMC6026206

[42] Papp H, László B, Jakab F, Ganesh B, De Grazia S, Matthijnssens J, et al. Review of group A rotavirus strains reported in swine and cattle. Vet Microbiol. 2013;165:190–9. 10.1016/j.vetmic.2013.03.020. 23642647 PMC7117210

[43] Brnić D, Čolić D, Kunić V, Maltar-Strmečki N, Krešić N, Konjević D, et al. Rotavirus A in Domestic Pigs and Wild Boars: High Genetic Diversity and Interspecies Transmission. Viruses. 2022;14:2028. 10.3390/v14092028. 36146832 PMC9503859

[44] Wenske O, Rückner A, Piehler D, Schwarz B-A, Vahlenkamp TW. Epidemiological analysis of porcine rotavirus A genotypes in Germany. Vet Microbiol. 2018;214:93–8. 10.1016/j.vetmic.2017.12.014. 29408039

[45] Monteagudo LV, Benito AA, Lázaro-Gaspar S, Arnal JL, Martin-Jurado D, Menjon R, et al. Occurrence of Rotavirus A Genotypes and Other Enteric Pathogens in Diarrheic Suckling Piglets from Spanish Swine Farms. Animals. 2022;12:251. 10.3390/ani12030251. 35158575 PMC8833434

[46] Salamunova S, Jackova A, Csank T, Mandelik R, Novotny J, Beckova Z, et al. Genetic variability of pig and human rotavirus group A isolates from Slovakia. Arch Virol. 2020;165:463–70. 10.1007/s00705-019-04504-6. 31863266

[47] Theuns S, Heylen E, Zeller M, Roukaerts IDM, Desmarets LMB, Van Ranst M, et al. Complete Genome Characterization of Recent and Ancient Belgian Pig Group A Rotaviruses and Assessment of Their Evolutionary Relationship with Human Rotaviruses. J Virol. 2015;89:1043–57. 10.1128/JVI.02513-14. 25378486 PMC4300624

[48] Silva FDF, Gregori F, McDonald SM. Distinguishing the genotype 1 genes and proteins of human Wa-like rotaviruses vs. porcine rotaviruses. Infect Genet Evol. 2016;43:6–14. 10.1016/j.meegid.2016.05.014. 27180895 PMC5119466

[49] Baumann S, Sydler T, Rosato G, Hilbe M, Kümmerlen D, Sidler X, et al. Frequent Occurrence of Simultaneous Infection with Multiple Rotaviruses in Swiss Pigs. Viruses. 2022;14:1117. 10.3390/v14051117. 35632858 PMC9147839

[50] Miyabe FM, Dall Agnol AM, Leme RA, Oliveira TES, Headley SA, Fernandes T, et al. Porcine rotavirus B as primary causative agent of diarrhea outbreaks in newborn piglets. Sci Rep. 2020;10:22002. 10.1038/s41598-020-78797-y. 33319798 PMC7738533

[51] Molinari BLD, Alfieri AF, Alfieri AA. Molecular Characterization of a New G (VP7) Genotype in Group B Porcine Rotavirus. Intervirology. 2018;61:42–8. 10.1159/000490388. 30011394

[52] Harima H, Qiu Y, Sasaki M, Ndebe J, Penjaninge K, Simulundu E, et al. A first report of rotavirus B from Zambian pigs leading to the discovery of a novel VP4 genotype P[9]. Virol J. 2024;21:263. 10.1186/s12985-024-02533-5. 39449113 PMC11515359

[53] Kim Y, Chang KO, Straw B, Saif LJ. Characterization of group C rotaviruses associated with diarrhea outbreaks in feeder pigs. J Clin Microbiol. 1999;37:1484–8. 10.1128/JCM.37.5.1484-1488.1999. 10203510 PMC84810

[54] Niira K, Ito M, Masuda T, Saitou T, Abe T, Komoto S, et al. Whole genome sequences of Japanese porcine species C rotaviruses reveal a high diversity of genotypes of individual genes and will contribute to a comprehensive, generally accepted classification system. Infect Genet Evol. 2016;44:106–13. 10.1016/j.meegid.2016.06.041. 27353186

[55] Kattoor JJ, Saurabh S, Malik YS, Sircar S, Dhama K, Ghosh S, et al. Unexpected detection of porcine rotavirus C strains carrying human origin VP6 gene. Vet Q. 2017;37:252–61. 10.1080/01652176.2017.1346849. 28643555

[56] Euring B, Harzer M, Vahlenkamp TW. Extended analyses of rotavirus C (RVC) G-types and P-types reveal new cut-off value for the G-types and reclassification of strains. J Virol. 2025;99:e00049–25. 10.1128/jvi.00049-25. 40231817 PMC12090754

[57] Pang XL, Lee B, Boroumand N, Leblanc B, Preiksaitis JK, Yu Ip CC. Increased detection of rotavirus using a real time reverse transcription-polymerase chain reaction (RT-PCR) assay in stool specimens from children with diarrhea. J Med Virol. 2004;72:496–501. 10.1002/jmv.20009. 14748075

[58] The Galaxy Community, Abueg LAL, Afgan E, Allart O, Awan AH, Bacon WA, et al. The Galaxy platform for accessible, reproducible, and collaborative data analyses: 2024 update. Nucleic Acids Res. 2024;52:W83–94. 10.1093/nar/gkae410. 38769056 PMC11223835

[59] Kolmogorov M, Bickhart DM, Behsaz B, Gurevich A, Rayko M, Shin SB, et al. metaFlye: scalable long-read metagenome assembly using repeat graphs. Nat Methods. 2020;17:1103–10. 10.1038/s41592-020-00971-x. 33020656 PMC10699202

[60] Li H. Minimap2: pairwise alignment for nucleotide sequences. Bioinformatics. 2018;34:3094–100. 10.1093/bioinformatics/bty191. 29750242 PMC6137996

[61] Katoh K, Standley DM. MAFFT Multiple Sequence Alignment Software Version 7: Improvements in Performance and Usability. Mol Biol Evol. 2013;30:772–80. 10.1093/molbev/mst010. 23329690 PMC3603318

[62] Zou Y, Zhang Z, Zeng Y, Hu H, Hao Y, Huang S, et al. Common Methods for Phylogenetic Tree Construction and Their Implementation in R. Bioengineering. 2024;11:480. 10.3390/bioengineering11050480. 38790347 PMC11117635

[63] Letunic I, Bork P. Interactive Tree of Life (iTOL) v6: recent updates to the phylogenetic tree display and annotation tool. Nucleic Acids Res. 2024;268. 10.1093/nar/gkae268. PMC11223838 38613393

[64] Pickett BE, Greer DS, Zhang Y, Stewart L, Zhou L, Sun G, et al. Virus Pathogen Database and Analysis Resource (ViPR): A Comprehensive Bioinformatics Database and Analysis Resource for the Coronavirus Research Community. Viruses. 2012;4:3209–26. 10.3390/v4113209. 23202522 PMC3509690

[65] Heberle H, Meirelles GV, da Silva FR, Telles GP, Minghim R. InteractiVenn: a web-based tool for the analysis of sets through Venn diagrams. BMC Bioinformatics. 2015;16:169. 10.1186/s12859-015-0611-3. 25994840 PMC4455604

