# Supplementary material for: High genetic diversity of porcine rotavirus A, B, and C in Hungary with putative novel VP4 genotypes
Source: BMC Vet Res. 2026 Jun 19;22:506. doi: 10.1186/s12917-026-05649-8 (PMC13527926; doi:10.1186/s12917-026-05649-8)
Supplement: Supplementary file 1 [file 12917_2026_5649_MOESM1_ESM.zip › 12917_2026_5649_MOESM1_ESM.pdf]

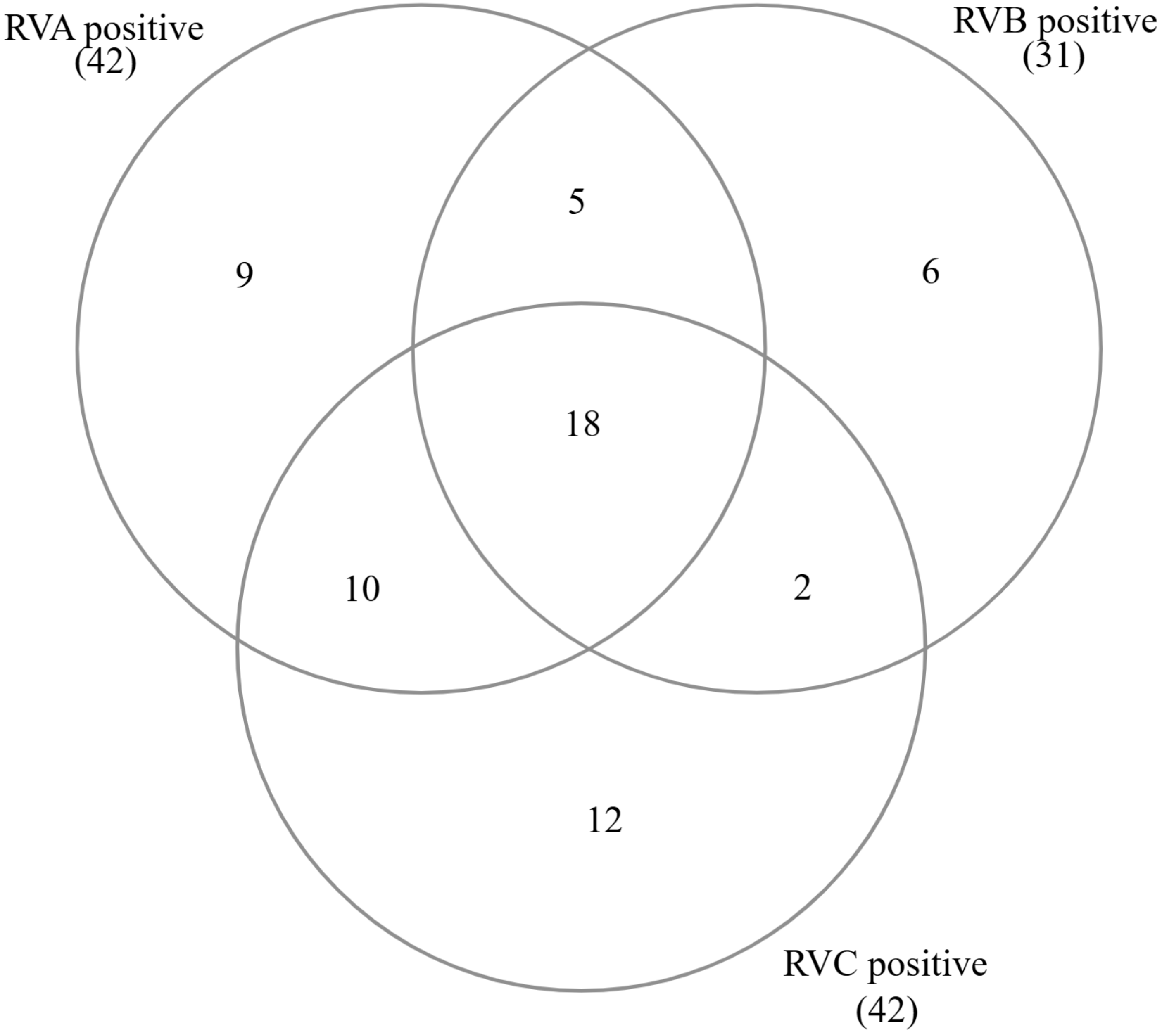
RVA positive
(42)
RVB positive
(31)
RVC positive
(42)
9
5
6
18
10
2
12
