# Supplementary material for: High genetic diversity of porcine rotavirus A, B, and C in Hungary with putative novel VP4 genotypes
Source: BMC Vet Res. 2026 Jun 19;22:506. doi: 10.1186/s12917-026-05649-8 (PMC13527926; doi:10.1186/s12917-026-05649-8)
Supplement: Supplementary file 1 [file 12917_2026_5649_MOESM1_ESM.zip › 12917_2026_5649_MOESM2_ESM.pdf]

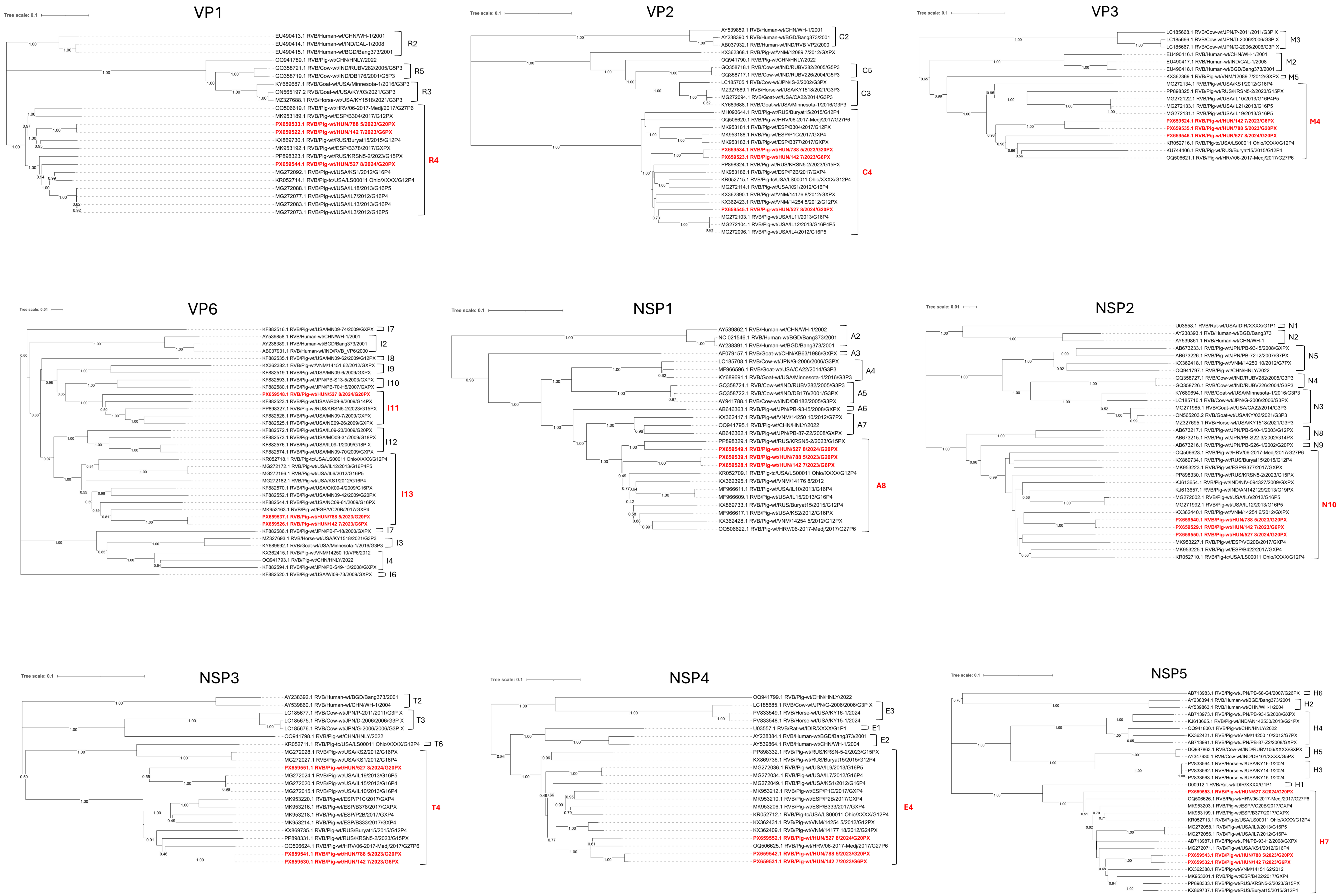

VP1
Tree scale: 0.1
R2
R5
R3
R4
VP2
Tree scale: 0.1
C2
C5
C3
C4
VP3
Tree scale: 0.1
M3
M2
M5
M4
VP6
Tree scale: 0.01
I7
I2
I8
I9
I10
I11
I12
I13
I3
I4
I6
NSP1
Tree scale: 0.1
A2
A3
A4
A5
A6
A7
A8
NSP2
Tree scale: 0.01
N1
N2
N5
N4
N3
N8
N9
N10
NSP3
Tree scale: 0.1
T2
T3
T6
T4
NSP4
Tree scale: 0.1
E3
E1
E2
E4
NSP5
Tree scale: 0.1
H6
H2
H4
H5
H3
H1
H7
