# Supplementary material for: High genetic diversity of porcine rotavirus A, B, and C in Hungary with putative novel VP4 genotypes
Source: BMC Vet Res. 2026 Jun 19;22:506. doi: 10.1186/s12917-026-05649-8 (PMC13527926; doi:10.1186/s12917-026-05649-8)
Supplement: Supplementary file 1 [file 12917_2026_5649_MOESM1_ESM.zip › 12917_2026_5649_MOESM3_ESM.pdf]

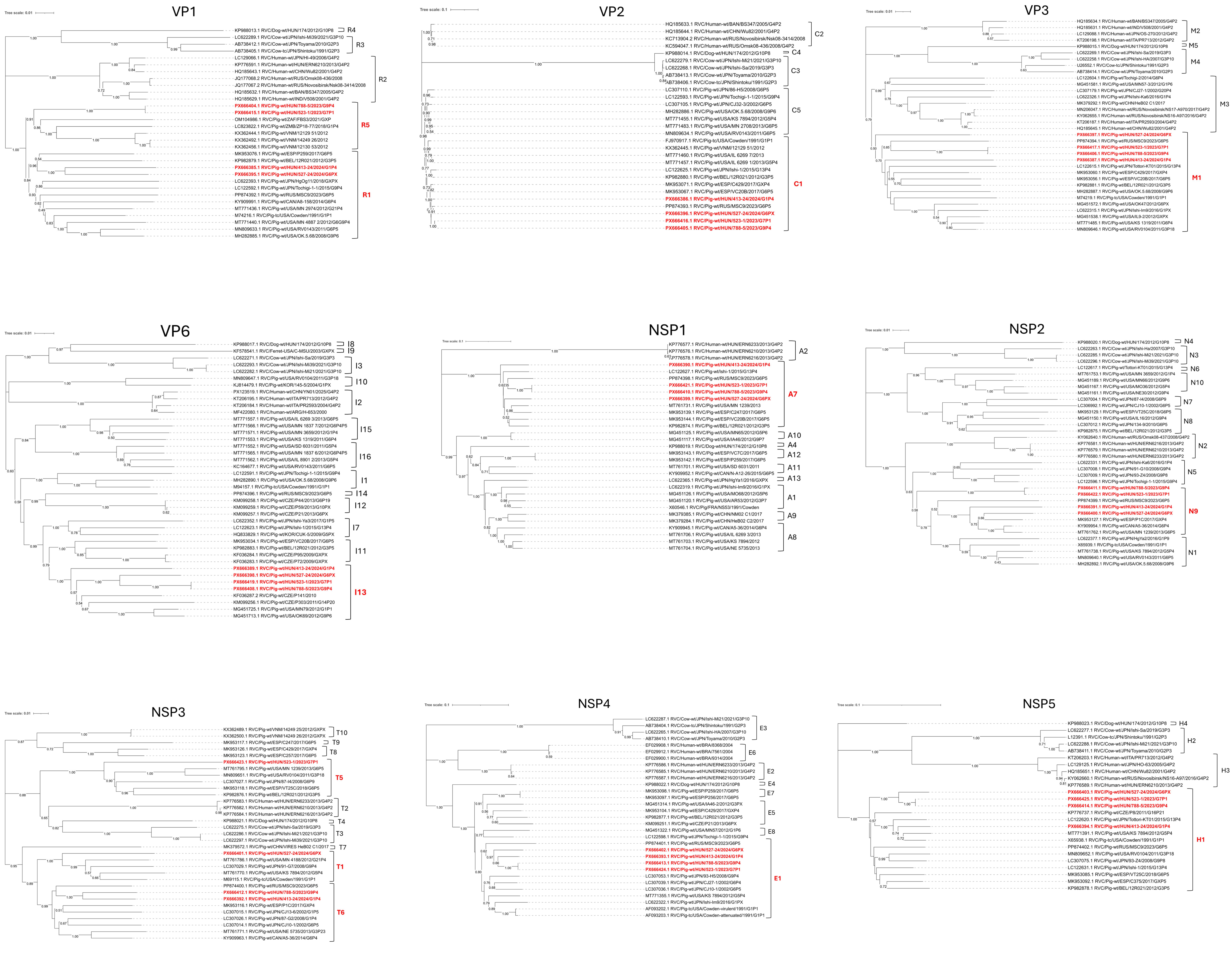
VP1
R4
R3
R2
R5
R1
VP2
C2
C4
C3
C5
C1
VP3
M2
M5
M4
M3
M1
VP6
I8
I9
I3
I10
I2
I15
I16
I1
I14
I12
I7
I11
I13
NSP1
A2
A7
A10
A4
A12
A11
A13
A1
A9
A8
NSP2
N4
N3
N6
N10
N7
N8
N2
N5
N9
N1
NSP3
T10
T9
T8
T5
T2
T4
T3
T7
T1
T6
NSP4
E3
E6
E2
E4
E7
E5
E8
E1
NSP5
H4
H2
H3
H1
