# Supplementary material for: High genetic diversity of porcine rotavirus A, B, and C in Hungary with putative novel VP4 genotypes
Source: BMC Vet Res. 2026 Jun 19;22:506. doi: 10.1186/s12917-026-05649-8 (PMC13527926; doi:10.1186/s12917-026-05649-8)
Supplement: Supplementary file 1 [file 12917_2026_5649_MOESM1_ESM.zip › 12917_2026_5649_MOESM4_ESM.pdf]

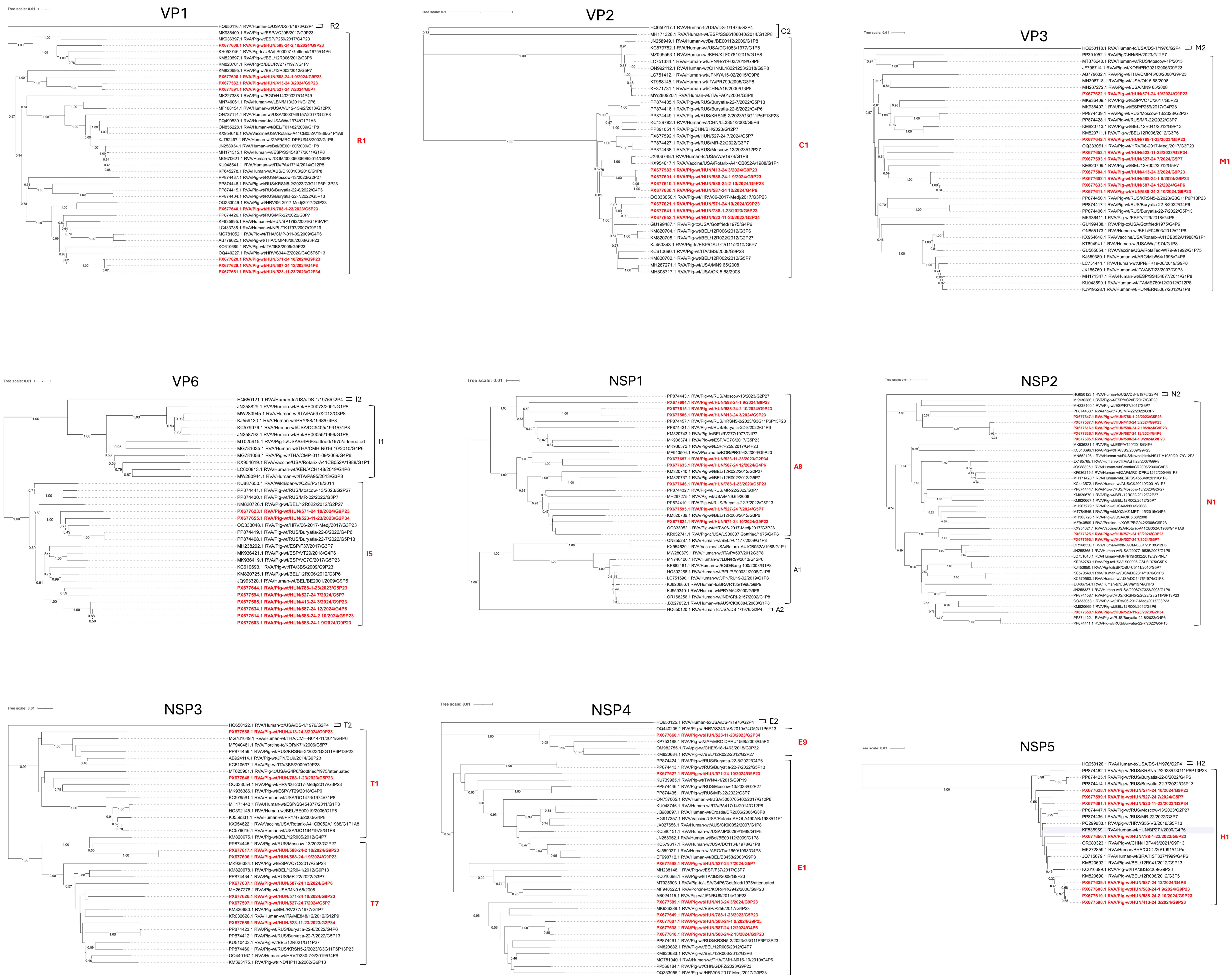
VP1
R2
R1
VP2
C2
C1
VP3
M2
M1
VP6
I2
I1
I5
NSP1
A8
A1
A2
NSP2
N2
N1
NSP3
T2
T1
T7
NSP4
E2
E9
E1
NSP5
H2
H1
